# Detection of PrP^res^ in peripheral tissue in pigs with clinical disease induced by intracerebral challenge with sheep-passaged bovine spongiform encephalopathy agent

**DOI:** 10.1371/journal.pone.0199914

**Published:** 2018-07-05

**Authors:** Carlos Hedman, Alicia Otero, Jean-Yves Douet, Caroline Lacroux, Séverine Lugan, Hicham Filali, Fabien Corbière, Naima Aron, Juan José Badiola, Olivier Andréoletti, Rosa Bolea

**Affiliations:** 1 Centro de Encefalopatías y Enfermedades Transmisibles Emergentes (CEETE), Veterinary Faculty, Universidad de Zaragoza, Zaragoza, Spain; 2 UMR INRA ENVT 1225, Interactions Hôtes Agents Pathogènes, Ecole Nationale Vétérinaire de Toulouse, Toulouse, France; University of Verona, ITALY

## Abstract

Bovine spongiform encephalopathy (BSE) can be efficiently transmitted to pigs via intracerebral inoculation. A clear link has been established between the consumption of products of bovine origin contaminated with the BSE agent and the development of variant Creutzfeldt-Jakob disease in humans. Small ruminants can also naturally develop BSE, and sheep-adapted BSE (Sh-BSE) propagates more efficiently than cattle BSE in pigs and in mouse models expressing porcine prion protein. In addition, Sh-BSE shows greater efficiency of transmission to human models than original cow BSE. While infectivity and/or abnormal PrP accumulation have been reported in the central nervous system in BSE-infected pigs, the ability of the agent to replicate in peripheral tissues has not been fully investigated. We previously characterized the presence of prions in a panel of tissues collected at the clinical stage of disease from pigs experimentally infected with Sh-BSE. Western blot revealed low levels of PrP^res^ accumulation in lymphoid tissues, nerves, and skeletal muscles from 4 of the 5 animals analysed. Using protein misfolding cyclic amplification (PMCA), which we found to be 6 log fold more sensitive than direct WB for the detection of pig BSE, we confirmed the presence of the Sh-BSE agent in lymphoid organs, nerves, ileum, and striated muscles from all 5 inoculated pigs. Surprisingly, PrP^res^ positivity was also detected in white blood cells from one pig using this method. The presence of infectivity in lymphoid tissues, striated muscles, and peripheral nerves was confirmed by bioassay in bovine PrP transgenic mice. These results demonstrate the ability of BSE-derived agents to replicate efficiently in various peripheral tissues in pigs. Although no prion transmission has been reported in pigs following oral BSE challenge, our data support the continuation of the Feed Ban measure implemented to prevent entry of the BSE agent into the feed chain.

## Introduction

Transmissible spongiform encephalopathies (TSE), or prion diseases, are progressive neurodegenerative disorders caused by the accumulation in the central nervous system (CNS) of PrP^Sc^ (or PrP^res^), an abnormal isoform of the cellular prion protein (PrP^C^) [1]. While a substantial transmission barrier usually limits interspecies propagation of prions, the agent of BSE has shown an uncommon ability to propagate in other species. Since the BSE epidemics in cattle [2], naturally occurring cases have been reported in a variety of zoo animals [3] and in farmed goats [4–6]. BSE was also responsible for the emergence in humans of variant Creutzfeldt-Jakob disease (vCJD), associated with the consumption of bovine products contaminated with BSE prions [3, 7].

To prevent recurrence of this type of event, the concept of specific risk material (SRM) was established, and many countries prohibited the inclusion of ruminant proteins in feed produced for cattle and other mammals. Moreover, several studies identified tissues outside the CNS in which PrP^res^ can be found. For example, in naturally infected cattle, prions have been detected mainly in the brain, spinal cord, retina, and distal ileum [8–11]. In sheep with experimentally-induced BSE, PrP^res^ has been detected in the lymphoreticular system (LRS) [12], various portions of the digestive tract, and in some components of the peripheral nervous system (PNS) [13, 14]. Peripheral accumulation of the BSE agent is thus more widespread in sheep than in cattle, although BSE can be transmitted to other species without obvious alterations in its neuropathological and biochemical features [7, 15, 16].

While pigs are susceptible to infection with the BSE agent following experimental parenteral inoculation [17–19], a strong transmission barrier has been described [20], and no cases of natural TSE infections have been reported in pigs. In a transgenic mouse model expressing porcine prion protein, the BSE agent can cross the cattle-pig transmission barrier more efficiently after passage in sheep (Sh-BSE) [21]. Furthermore, we have shown that Sh-BSE prions propagate efficiently in intracerebrally inoculated pigs, and that PrP^res^ accumulation can be detected by enzyme immunoassay and immunohistochemistry in a wide variety of peripheral tissues from these animals [22]. However, conventional assays offer limited sensitivity, and may fail to detect prions in tissues and fluids in which PrP^res^ is present at very low levels. Here we describe a comparative protein misfolding cyclic amplification (PMCA) assay and mouse bioassay for the detection of PrP^res^ in a panel of tissues collected from pigs intracerebrally inoculated with Sh-BSE.

To characterize the distribution of PrP^res^ during clinical disease, we analysed different tissues from Sh-BSE-inoculated pigs exhibiting clinical signs. Mouse bioassay revealed PrP^res^ infectivity in the CNS, PNS, skeletal muscle, and mesenteric lymph nodes. Moreover, the PMCA technique allowed further amplification of PrP^res^ in ileum, spleen, and white blood cell (WBC) samples collected from Sh-BSE-inoculated pigs displaying clinical signs of disease.

## Materials and methods

### Ethics statement

All animal experiments were performed in strict accordance with European Community Council Directive 86/609/EEC for the protection of animals used for experimental and other scientific purposes and were approved by the relevant local ethics committees. The INRA/Toulouse ENVT Ethics Committee approved the experimental protocol in mice.

### Sh-BSE in pig experimental cases

Tissue samples used in the present study were obtained from five minipigs that were experimentally infected by intracerebral inoculation with the Sh-BSE agent [22]. These animals were challenged with 0.5 ml of a 10% brain pool obtained from BSE experimentally infected sheep [23]. Intracerebral injections were performed through the frontal bone using a trephine and a 20G × 2 ¾” needle and were carried out under general anesthesia. Sh-BSE challenged pigs were monitored daily by animal husbandry staff, and veterinary clinical assessments were performed weekly in order to evaluate their clinical status and determine the appearance of signs that could seriously compromise the welfare of the animals. All challenged pigs showed clinical signs of TSE between 77 and 109 weeks post inoculation, and the clinical picture was characterized by early behavioral changes, followed by locomotor disability with progressive ataxia and weakness. Animals were euthanized by intravenous pentobarbital injection (DOLETHAL^®^; 10 mg/kg) followed by exsanguination when they showed any impairment in their ability to stand up or any welfare-compromising clinical sign [22]. Details of individual incubation periods, clinical signs and brain PrP^Sc^ detection are described in Hedman et al., 2016.

### Preparation of inocula

Samples of 7 tissue types (brain, brachial nerve, sciatic nerve, mesenteric lymph node, oculomotor muscle, ileum, and spleen) were collected from Sh-BSE-infected pigs [22] for PMCA and mouse bioassays. In each case, disposable equipment (forceps and scalpels) was used to manipulate the tissues, which were collected under TSE sterile conditions, precluding cross contamination during sample preparation. Tissue inocula were prepared in sterile saline solution (1:10) in single-use micro tubes (Precess 48, BioRad) and were filtered using a syringe. Tissue homogenates were then aliquoted and stored at -80°C. Peripheral tissues and CNS homogenates were prepared separately. Tissues from an uninfected pig were used as negative controls.

Before sacrifice, whole blood was collected from the jugular vein of Sh-BSE-infected pigs, using 10-ml tubes containing EDTA (ethylenediaminetetraacetic acid). Each tube was centrifuged continuously at room temperature for 15 min at 1,300 × g. The buffy coat fraction was collected using a disposable hard-bulb pipette and mixed with 6 ml of PBS (phosphate buffered saline). This mixture was then deposited over 3 ml of Lymphoprep solution (STEMCELL Technologies) and centrifuged at 1,300 × g for 15 min. The WBCs obtained were then washed 3 times in PBS and aliquoted before freezing (-80°C) and were subsequently analysed for prion seeding activity and infectivity by PMCA and mouse bioassay, respectively.

### Protein misfolding cyclic amplification

Mouse brain lysate from uninfected TgARQ mice was used as substrate for amplification of Sh-BSE in pig prions. After sacrifice by CO_2_ exposure, mouse brains were rapidly removed and washed twice in Ca^++^/Mg^++^ free PBS containing 5 mM EDTA. Brains were either used immediately to prepare the PrP^C^ substrate lysate or stored at -80°C. The PrP^C^ substrate (10% brain lysate) was prepared using a Dounce tissue grinder to completely homogenize the brain tissue in cold PMCA buffer (50 mM Tris-HCl, pH 7.4, 5 mM EDTA, 300 mM NaCl, 1% Triton X-100). The substrate was left at 4°C for 30 min, and then aliquoted and stored at -80°C.

PMCA was performed by mixing 5 μl of seed with 45 μl of substrate per well in a 96-well microplate (Axygen, Union City, CA, USA). One teflon bead (diameter, 2.381 mm) was added to each well. PMCA seeds were serially diluted 10^−1^ to 10^−11^ fold in conversion buffer (final volume, 100 μl). Next, 5 μl from the previous dilution was added to each well, which contained 45 μl of substrate. Microplates were placed in a sonicator (Misonix 4000) and subjected to 96 cycles of sonication (30 s of sonication, 30-min incubation at 37°C) at 70% power. Three subsequent rounds of PMCA were performed using a 1/10 dilution of the products of the previous PMCA round as the template. On completion of the PMCA procedure, aliquots from each sample were collected for analysis of PrP^res^ content.

### Dot blot detection of PrP^res^

To determine the detection limit and sensitivity of the PMCA method used to amplify pig Sh-BSE PrP^res^, dot blot was used to analyse the presence of PrP^res^ in a 10-fold dilution series of brain homogenate from one pig after 3 rounds of PMCA.

Each PMCA product (18 μl) was supplemented with 3% SDS and treated with proteinase K (PK) (final concentration, 50μg/ml) for 1 h at 37°C. Digestion was stopped by adding an equal volume of Laemmli buffer and heating at 100°C for 5 min. A 5-μl volume of sample was mixed with 25 μl of 1% SDS. The samples were then vacuum transferred onto a nitrocellulose membrane. The membrane was rinsed once with PBS (0.1% Tween 20) and incubated for 30 min in PBS containing 2% BSA. The monoclonal antibody Sha31 (1:8000 in PBS+2% BSA), which recognize residues 145–152 (YEDRYYRE) of PrP, was used for PrP^res^ immunodetection [24]. Antibody binding was detected by incubating the membranes for 20 min with conjugated mouse anti-goat IgG (1:5000; Santa Cruz Biotechnology). Immunoblots were developed by enhanced chemiluminescence using ECL reagent (Pierce) and visualized using the Versa Doc Quantity One image analysis system (Bio-Rad).

### Bioassay

The mouse bioassay was performed in bovine PrP transgenic mice (tgBov/tg110) expressing bovine PrP^C^ at levels 8-fold higher than those detected in cow brain. TgBov mice are considered a highly efficient model for the detection of BSE infectivity [25]. At least 6 mice were intracerebrally inoculated with each sample (20 μl). Mice were clinically monitored for the onset of clinical signs of murine prion disease. Mice were euthanized when neurological dysfunction was evident, and they started to show locomotor disorders and/or any limitation in their ability to feed. CNS and spleen samples were individually collected and subsequently analysed for PrP^res^.

### Western blot detection of PrP^res^

The TeSeE Western blot kit (Bio-Rad) was used following the manufacturer’s recommendations. Samples taken from the brain, sciatic nerve, brachial nerve, mesenteric lymph node, oculomotor muscle, ileum, and spleen, and WBCs obtained from Sh-BSE-challenged pigs, were analysed for the presence of PrP^res^. For PMCA products, 20 μl of reaction product was mixed with 230 μl of 10% negative brain homogenate before PrP^res^ extraction, as previously described [26]. PrP^res^ detection was performed using Sha31 mAb conjugated to horseradish peroxidase (0.06 μg/ml). ECL substrate (Pierce) was used to reveal peroxidase activity.

## Results

**Fig 1:**
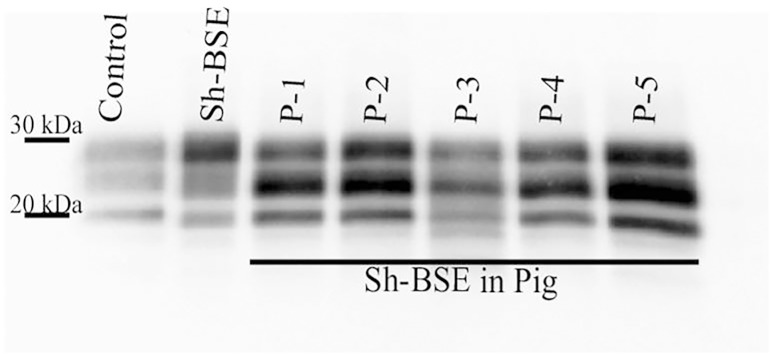
Western-blot analysis to detect the proteinase K-resistant core fragment (PrP^res^) of the pathologic prion in the brain of 5 pigs experimentally infected with Sh-BSE. PrP^res^ western-blot profile from the original Sh-BSE isolate, characterized by predominance of the diglycosylated band, is shown for comparison. Immunodetection was performed using the monoclonal Sha31 antibody. A PK digested classical scrapie isolate (Dawson strain) was used as positive control (Control).

**Fig 2:**
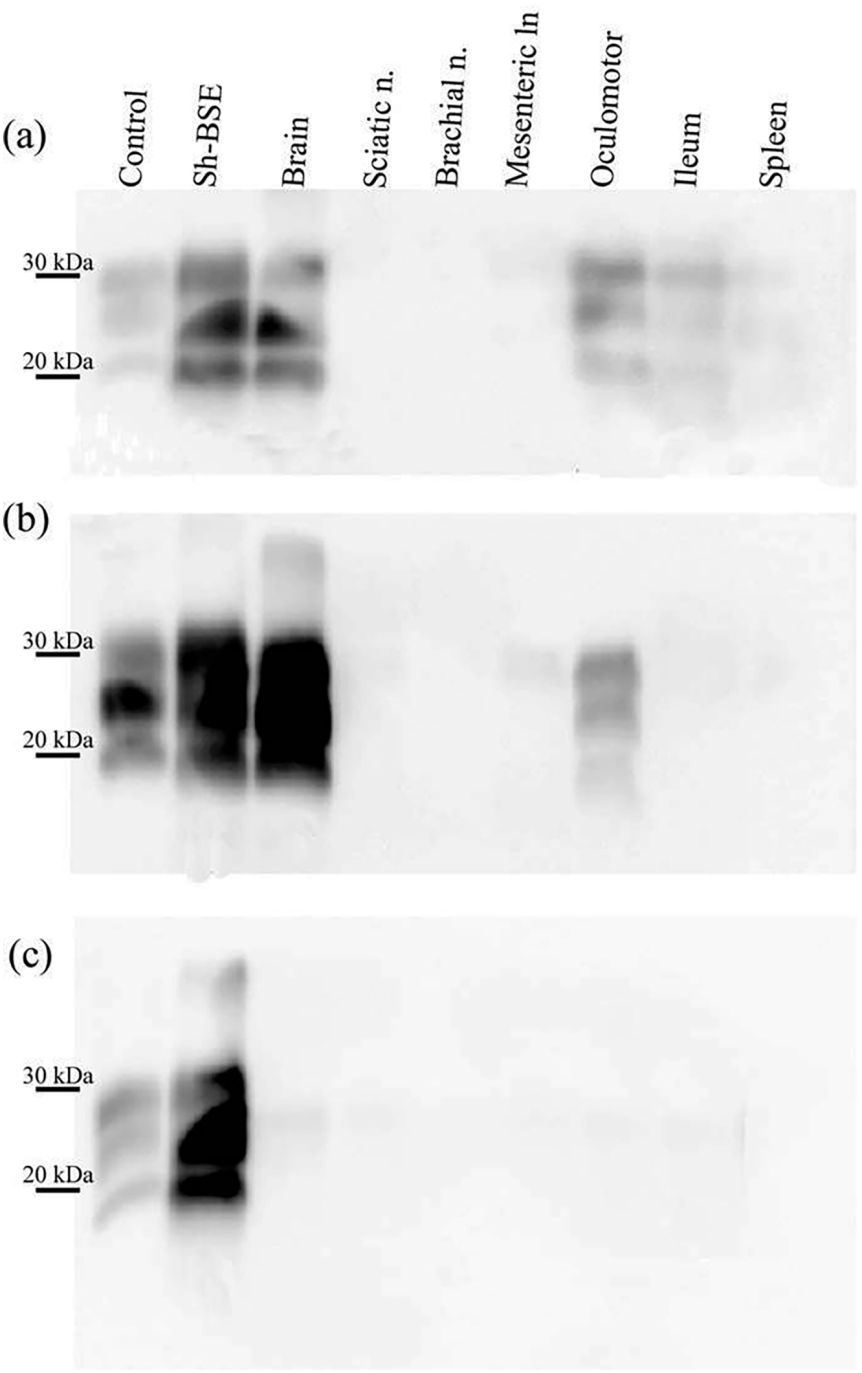
Detection of PrP^res^ in the brain and peripheral tissues of Sh-BSE-inoculated pigs with clinical disease. (a) A strong PrP^res^ signal is detected in the brain of one pig sacrificed at 17 months post inoculation (mpi), whereas a much weaker signal is observed in oculomotor muscle and ileum samples from the same animal. (b) PrP^res^ detection in brain and oculomotor muscle samples from a pig sacrificed at 30 mpi. (c) No PrP^res^ was detected in tissues from the negative control pig. Immunodetection was performed using the monoclonal Sha31 antibody. Control: PK digested classical scrapie isolate.

Using Western blot, we demonstrated PrP^res^ positivity in the brains of all Sh-BSE-inoculated pigs (Fig 1). The animals analysed in the present study were euthanized at 17, 26, and 27 months post-inoculation (mpi), and 2 more at 30 mpi (S1 Table) [22]. In some pigs, a positive PrP^res^ signal was observed in specific peripheral tissues, such as the oculomotor muscle and ileum (Fig 2a and 2b). However, in others, no PrP^res^ signal was detected in any of the peripheral tissues analysed (Table 1). No PrP^res^ was detected in tissue samples from the negative control pig (Fig 2c). In all tissues analysed, we detected a predominance of the monoglycosylated PrP^res^ fraction (Figs 1 and 2); the WB electrophoretic pattern was therefore identical to that previously described in pigs, and similar to that described in transgenic mice expressing a pig PrP amino-acid sequence [27].

**Table 1:** Western blot detection of PrP^res^ in the original inocula prepared with tissues collected from Sh-BSE-inoculated pigs with clinical disease.

ID	Brain	Sciatic nerve	Brachial nerve	Mesenteric lymph node	Exocular muscle	Ileum	Spleen	WBC
**P-0**	-	-	-	-	-	-	-	ND
**P-1**	+	-	-	-	+	+	-	ND
**P-2**	+	-	-	-	-	-	-	ND
**P-3**	+	+	-	-	+	+	+	ND
**P-4**	+	-	-	-	-	+	-	ND
**P-5**	+	-	-	-	+	-	-	ND

Negative:—Positive: + No determined: ND

Since the PrP^res^ Western blot method is of limited sensitivity, we performed *in vitro* amplification by PMCA. This method allows amplification of minimal amounts of prion and has been reported to be an efficient means of detecting the BSE agent in blood from vCJD patients [28]. To assess the utility of this method for amplifying pig Sh-BSE and minute quantities of PrP^res^, a 10-fold dilution series of brain homogenate (from P-1) was subjected to 3 successive rounds of PMCA using brain homogenate from transgenic mice expressing the ovine ARQ PrP as a substrate. After one round of PMCA, PrP^res^ was detected in reactions seeded with a 10^−3^ dilution of the reference pig isolate. After 3 rounds of PMCA, PrP^res^ was detected in reactions seeded with a 10^−9^ dilution of the brain homogenate (S1 Fig). By contrast, the highest dilution of brain homogenate for which a positive PrP^res^ signal was detected by WB was the 10^−3^ dilution (S2 Fig). These results indicate that the PMCA method used is approximately 6 log10-fold more sensitive than direct WB for the detection of pig Sh-BSE prions.

**Fig 3:**
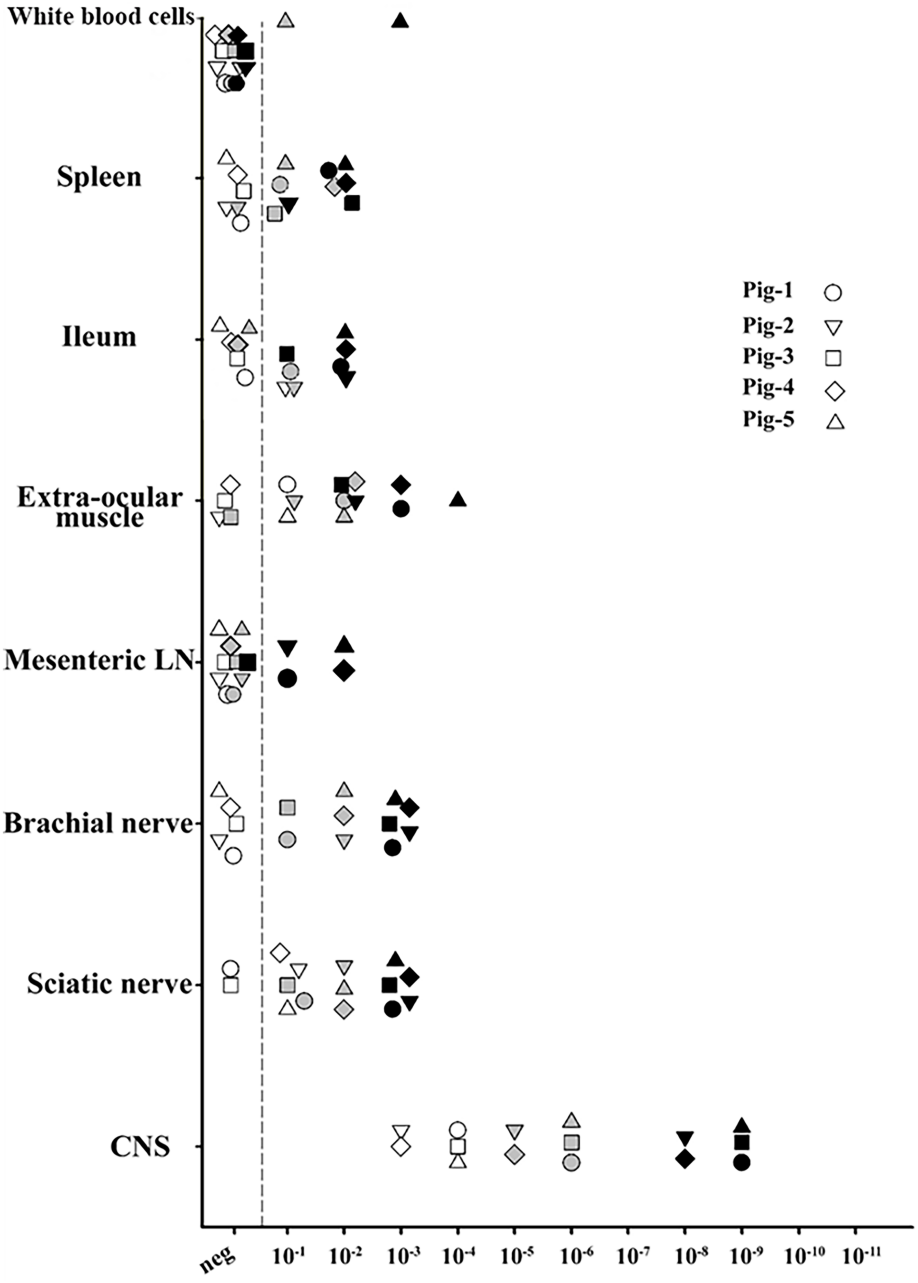
Relative performance of protein misfolding cyclic amplification of porcine Sh-BSE using brain tissue from ARQ/ARQ ovine PrP transgenic mice as substrate. PMCA reactions were seeded with a 10-fold dilution series (10^−1^–10^−11^) of 8 different tissues collected from 5 Sh-BSE-infected pigs. For each tissue, the last PrP^res^-positive dilution as detected by Western blot after 1 (white), 2 (grey), and 3 (black) consecutive amplification rounds (96 cycles in a Misonix 4000 sonicator) is shown. The following tissues were assayed: brain, sciatic nerve, brachial nerve, mesenteric lymph node, extraocular muscle, ileum, spleen, and white blood cells.

**Fig 4:**
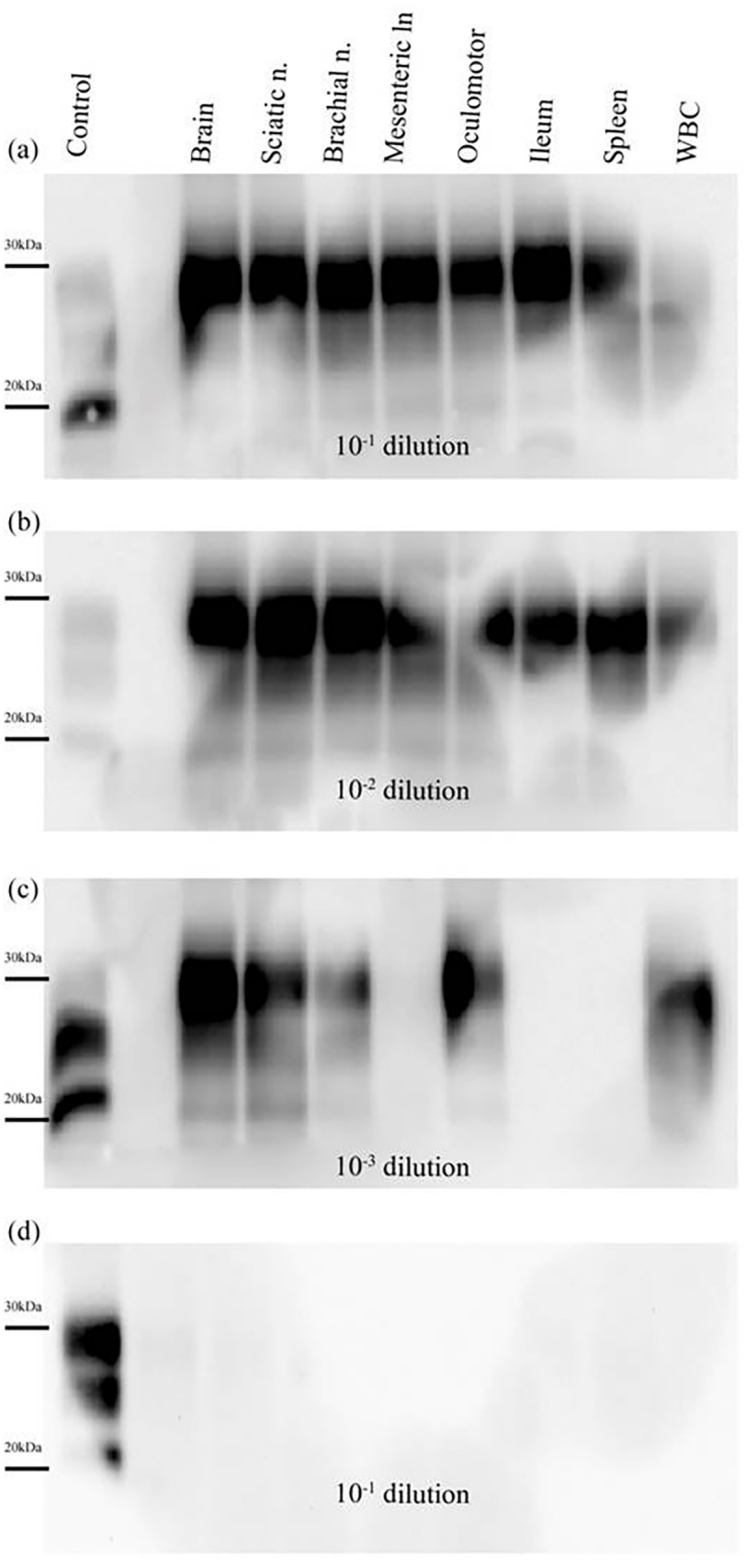
PrP^res^ detection in PMCA reactions seeded with tissue samples collected from a Sh-BSE clinically affected pig. Tissues: brain, sciatic nerve, brachial nerve, mesenteric lymph node, oculomotor muscle, ileum, spleen, and white blood cells (WBC). The animal was culled at 30 months after intracerebral inoculation with Sh-BSE. After 3 rounds of PMCA (48 hours each), PrP^res^ amplification was detected at dilutions of 10^−1^ (a) and 10^−2^ (b) in all the tissues analysed. No PrP^res^ was amplified in the mesenteric lymph node, ileum, or spleen at a dilution of 10^−3^ (c). Results obtained for 10^−1^ dilution after 3 rounds of PMCA in reactions seeded with tissues from a negative control pig (d). Control: PK digested classical scrapie isolate.

After 3 rounds of PMCA, PrP^res^ was detected in reactions seeded with all of the tissue samples, except for the mesenteric lymph node sample from P-3 (Fig 3). Unexpectedly, reactions seeded with WBCs obtained from P-5 were also positive. (Figs 3 and 4a–4c). No PrP^res^ was detected in reactions seeded with tissue homogenates from the negative control pig, confirming the specificity of the technique. (Fig 4d). Based on the detection limit of PMCA after 3 rounds, seeding activity in peripheral tissues was between 8 log10 and 4 log10 lower than that detected in brain tissue (Fig 3).

Parallel to PMCA, tissue homogenates from P-1 were also tested for infectivity by bioassay in transgenic mice expressing bovine PrP (tgBov mice). The results confirmed the presence of the Sh-BSE agent in the PNS, mesenteric lymph nodes, and oculomotor muscle in clinically affected pigs. We observed lower attack rates (<100%) and/or more prolonged incubation periods in tgBov mice inoculated with peripheral tissues than those inoculated with brain homogenate. These results are consistent with the substantially lower infectivity in those tissues than in the CNS (Table 2). No clinical disease or PrP^res^ accumulation were observed in mice inoculated with spleen, ileum, or WBC samples from P-1, or in mice inoculated with control brain homogenate.

**Table 2:** Intracerebral inoculation of tgBov mice with a panel of tissues collected from a Sh-BSE clinically affected pig.

Inocula	Brain	Sciatic nerve	Brachial nerve	Mesenteric lymph node	Oculomotor muscle	Ileum	Spleen	WBC
**Positive mice**^a^	5/5	5/6	2/6	1/5	5/5	0/5	0/5	0/5
**Incubation**^b^	306±85	402± 76	478±0	477	412 ±86	-	-	-

^a^ Data based on PrP^res^ detection in the brain

^b^ Incubation periods were calculated as the number of days between inoculation and the onset of clinical signs consistent with a prion disease, at which time mice were euthanized. Incubation periods are shown as mean±SEM days post-inoculation

## Discussion

In the present study, we used a modified PMCA amplification protocol [29], and a mouse infectivity bioassay to examine the distribution of prions in peripheral tissue in 5 pigs following intracerebral challenge with sheep-passaged BSE agent [22].

In the 5 challenged pigs, PrP^res^ accumulation in the CNS was confirmed by WB (Fig 1). In line with all published BSE transmission experiments in pigs or transgenic mice expressing pig PrP^C^ [20–22, 30], the PrP^res^ WB pattern revealed predominance of the monoglycosylated PrP^res^ moiety. In peripheral tissues, some variation in PrP^res^ detection was observed between animals. Direct WB revealed the presence of PrP^res^ in oculomotor muscle and ileum samples from a pig sacrificed at 17 mpi, but only in oculomotor muscle in a pig that succumbed to disease at 30 mpi (Fig 2). These results suggest that, in Sh-BSE clinically affected pigs, there is no apparent correlation between the incubation period and peripheral PrP^Sc^ dissemination. After 3 rounds of PMCA, some degree of variation in seeding activities in peripheral tissues was also observed between pigs (Fig 3 and S1 Table). Moreover, PrP^res^ in peripheral tissues was detected in reactions seeded with tissue homogenates diluted at 10^−1^ to 10^−4^, but in reactions seeded with brain homogenate at dilutions of 10^−8^–10^−9^ (S1 Fig). Thus, in Sh-BSE infected pigs, the accumulation of prions was significantly greater in the brain than in peripheral tissues, in which PrP^res^ accumulation was more restricted, and varied considerably between animals. This heterogeneous accumulation of PrP^res^ in peripheral tissues may be partially explained by tissue sampling, since there is a reported variation in PrP^res^ concentration between different tissue regions depending on the absence or presence of embedded nerve endings or individual lymphoid follicles, as described in cattle with BSE [31, 32].

No false positive reactions were observed, and the specificity of the technique was demonstrated by the absence of detectable PrP^res^ after 3 rounds of PMCA of a diluted sample of brain homogenate from an uninfected pig (Fig 4d). As mentioned above, the PMCA protocol used allowed detection of PrP^res^ after 3 rounds in reactions seeded with a 10^−9^ dilution of brain homogenate, a similar sensitivity to that previously described, using the same protocol, for pig adapted BSE and other BSE-derived strains [28].

Bioassays were performed using tgBov mice, which are highly susceptible to propagation of the BSE agent [25]. Survival times for tgBov mice inoculated with brain homogenate from Sh-BSE-infected pigs (306 ± 81 dpi) were similar to those previously described for tgBov mice inoculated with pig-adapted BSE [27].

To our knowledge, this is the first report demonstrating the infectivity in a mouse bioassay of sciatic and brachial nerves, skeletal muscle (oculomotor), and mesenteric lymph nodes from pigs intracerebrally inoculated with BSE-derived agents. In a previous study, we evaluated the neuropathology and distribution of Sh-BSE prions in intracerebrally inoculated pigs with clinical disease. Using immunohistochemistry, we demonstrated the presence of Sh-BSE prions in the nervous components of a variety of peripheral tissues, including nerve fibres of the oculomotor muscle, the myenteric plexi of the gastrointestinal tract, retinal ganglion cells, and the postganglionic neurons of the adrenal glands and pancreas [22]. The specific location of PrP^Sc^ in these tissues, together with the results obtained in the present study, suggests a centrifugal spread of the agent from the CNS to the organs via the PNS. However, in one infected animal we also observed PrP^Sc^ deposition in the epithelial tubular cells and the collecting ducts of the kidney in the absence of nephritis [22]. This localization of PrP^Sc^ aggregates in renal tissues suggests the arrival PrP^Sc^ to this organ via the blood [33]. Given that we also detected prion seeding activity in WBCs from one animal, we cannot rule out the possibility that prions are also haematogenously disseminated in Sh-BSE-infected pigs. In previous studies in which the BSE agent was transmitted to pigs, mouse bioassay revealed infectivity in the CNS, stomach, jejunum, ileum, and pancreas of animals sacrificed in the terminal stage of the disease [17, 19, 34]. However, those animals were simultaneously inoculated via intracerebral, intraperitoneal, and intravenous routes. As such, no solid conclusions can be reached as to the spread of prions to these tissues, since the presence of infectivity could be the result of persistence of intraperitoneally injected inoculum [35] or local replication of the agent [36].

The low attack rates and/or extended incubation times observed in mice inoculated with brachial nerve, mesenteric lymph node, and ileum samples suggests a low titre of infectivity in these tissues. These results are consistent with the low conversion efficiency of PMCA reactions seeded with those same tissues as compared with that observed in reactions seeded with brain homogenate (Fig 3). In contrast to the negative results obtained in the bioassay, PMCA allowed detection of PrP^res^ in reactions seeded with a 10^−2^ dilution of spleen and ileum tissue from P-1. While neither seeding activity nor infectivity was observed for WBCs from P-1, PrP^res^ was detected in reactions seeded with WBCs from P-5 at a 10^−3^ dilution after 3 rounds of PMCA. Thus, these results represent an evidence for prionaemia [37] in pigs experimentally infected with BSE-derived agents. It has been previously demonstrated that prion diseases can be efficiently transmitted by blood transfusion [38], and that WBC are appropriate targets for prion detection in scrapie [39], BSE, and vCJD, even in preclinical disease stages [40, 41].

We have shown that Sh-BSE is efficiently transmitted to pigs after intracerebral inoculation, and that PrP^Sc^ in inoculated animals is widely distributed in a variety of peripheral tissues [22]. We show that PrP^res^ is also present in tissues in which prion deposition is not detected by conventional techniques and, for the first time, we demonstrate the detection of BSE-derived agents in the blood of prion-infected pigs. The ability of pig-adapted prions to accumulate in peripheral tissues and blood components may have important implications, given the broad range of pig-derived food products, being blood the main constituent of some of them. However, it is important to bear in mind the limitations associated with intracerebral inoculation, since the route of infection, together with *PRNP* genotype and prion strain is one of the key factors controlling the transmission barrier. Compared with intracerebral inoculation, infection via the oral route, which accounts for the majority of natural infections, is generally a much less efficient means of transmitting the disease [42]. In addition to the lack of natural TSE cases reported in pigs, attempts to orally transmit BSE to pigs have been unsuccessful [34]. However, it should be considered that Sh-BSE has been reported to have an increased pathogenicity compared to cattle BSE in studies using transgenic mice expressing porcine [21] or human PrP [43, 44]. In addition, it has been demonstrated that sheep infected with the BSE agent show a significantly wider tissue distribution of both PrP^Sc^ and infectivity than BSE-infected cattle [9, 11, 45–47]. In the present study, we have detected the Sh-BSE agent in a wide variety of extraneural tissues. Sh-BSE prions also seem to present a wider tisular spread than cattle BSE in the porcine species, [22], although both agents are intracerebrally transmitted to the pig with a similar incubation period [22, 34]. Therefore, despite infructuous attempts to orally transmit cattle BSE to pigs, it should not be ruled out that Sh-BSE could be transmitted to the porcine species via the oral route, and it should be considered that the agent may be present in a greater number of tissues. Considering that natural BSE cases have been described in small ruminants [6, 48] and given that Sh-BSE, as aforementioned, propagate more efficiently than cattle BSE in human PrP transgenic mice [43], we consider that further studies of oral exposure of pigs to Sh-BSE will be necessary to determine the potential risk of natural transmission in this species.

In summary, our results reinforce the view that despite a significant transmission barrier, BSE can replicate in the peripheral tissues of pigs exposed to BSE agent. Taken together with the results of previous studies, these data support the continuation of the Feed Ban implemented to prevent the entry of BSE agent into the feed chain.

## Supporting information

S1 Fig PrP^res^ detection by dot blot in PMCA reactions seeded with serially diluted (10^−1^–10^−10^) brain homogenate from a Sh-BSE-infected pig. After 3 rounds of PMCA, PrP^res^ was amplified in reactions seeded with a 10^−9^ dilution of a brain homogenate from the Sh-BSE-infected pig, demonstrating the sensitivity of the PMCA protocol used to detect pig Sh-BSE prions. The monoclonal Sha31 antibody was used for immunodetection.
(TIF)

S2 Fig Western-blot detection of PrP^res^ in serially diluted brain homogenates (10^−1^–10^−5^) from Sh-BSE-infected pigs. Western blot allowed detection of PrP^res^ present in dilutions (10^−1^–10^−3^) of the original homogenates before PMCA. The monoclonal Sha31 antibody was used for immunodetection. MM: Magic Marker.
(TIF)

S1 Table Individual survival periods and detection limits of Western blot and PMCA for brain samples of Sh-BSE inoculated pigs. (PDF)

## References

[1] Prusiner SB. Molecular biology of prion diseases. Science. 1991;252(5012):1515–22. . 1675487 10.1126/science.1675487

[2] Wells GA, Scott AC, Johnson CT, Gunning RF, Hancock RD, Jeffrey M, et al A novel progressive spongiform encephalopathy in cattle. Vet Rec. 1987;121(18):419–20. . 3424605 10.1136/vr.121.18.419

[3] Hill AF, Desbruslais M, Joiner S, Sidle KCL, Gowland I, Collinge J, et al The same prion strain causes vCJD and BSE. Nature. 1997;389(6650):448–50. doi: 10.1038/38925 9333232 10.1038/38925

[4] Eloit M, Adjou K, Coulpier M, Fontaine JJ, Hamel R, Lilin T, et al BSE agent signatures in a goat. Veterinary Record. 2005;156(16):523–4. 10.1136/vr.156.16.523-b 15833975

[5] Jeffrey M, Martin S, Gonzalez L, Foster J, Langeveld JP, van Zijderveld FG, et al Immunohistochemical features of PrP(d) accumulation in natural and experimental goat transmissible spongiform encephalopathies. J Comp Pathol. 2006;134(2–3):171–81. Epub 2006/03/18. doi: 10.1016/j.jcpa.2005.10.003 . 16542672 10.1016/j.jcpa.2005.10.003

[6] Spiropoulos J, Lockey R, Sallis RE, Terry LA, Thorne L, Holder TM, et al Isolation of prion with BSE properties from farmed goat. Emerg Infect Dis. 2011;17(12):2253–61. doi: 10.3201/eid1712.110333 . 22172149 10.3201/eid1712.110333 PMC3311188

[7] Bruce ME, Will RG, Ironside JW, McConnell I, Drummond D, Suttie A, et al Transmissions to mice indicate that ‘new variant’ CJD is caused by the BSE agent. Nature. 1997;389(6650):498–501. doi: 10.1038/39057 . 9333239 10.1038/39057

[8] Buschmann A, Groschup MH. Highly bovine spongiform encephalopathy-sensitive transgenic mice confirm the essential restriction of infectivity to the nervous system in clinically diseased cattle. Journal of Infectious Diseases. 2005;192(5):934–42. doi: 10.1086/431602 16088845 10.1086/431602

[9] Wells GA, Hawkins SA, Green RB, Austin AR, Dexter I, Spencer YI, et al Preliminary observations on the pathogenesis of experimental bovine spongiform encephalopathy (BSE): an update. Vet Rec. 1998;142(5):103–6. . 9501384 10.1136/vr.142.5.103

[10] Wells GA, Spiropoulos J, Hawkins SA, Ryder SJ. Pathogenesis of experimental bovine spongiform encephalopathy: preclinical infectivity in tonsil and observations on the distribution of lingual tonsil in slaughtered cattle. Vet Rec. 2005;156(13):401–7. . 15816193 10.1136/vr.156.13.401

[11] Espinosa JC, Morales M, Castilla J, Rogers M, Torres JM. Progression of prion infectivity in asymptomatic cattle after oral bovine spongiform encephalopathy challenge. J Gen Virol 2007;88:1379–83. doi: 10.1099/vir.0.82647-0 17374785 10.1099/vir.0.82647-0

[12] Andreoletti O, Morel N, Lacroux C, Rouillon V, Barc C, Tabouret G, et al Bovine spongiform encephalopathy agent in spleen from an ARR/ARR orally exposed sheep. J Gen Virol. 2006;87:1043–6. doi: 10.1099/vir.0.81318-0 16528056 10.1099/vir.0.81318-0

[13] Foster JD, Bruce M, McConnell I, Chree A, Fraser H. Detection of BSE infectivity in brain and spleen of experimentally infected sheep. Veterinary Record. 1996;138(22):546–8. 8782362 10.1136/vr.138.22.546

[14] Bellworthy SJ, Hawkins SA, Green RB, Blamire I, Dexter G, Dexter I, et al Tissue distribution of bovine spongiform encephalopathy infectivity in Romney sheep up to the onset of clinical disease after oral challenge. Vet Rec. 2005;156(7):197–202. Epub 2005/03/08. . 15747655 10.1136/vr.156.7.197

[15] Hill AF, Desbruslais M, Joiner S, Sidle KC, Gowland I, Collinge J, et al The same prion strain causes vCJD and BSE. Nature. 1997;389(6650):448–50, 526. doi: 10.1038/38925 . 9333232 10.1038/38925

[16] Torres JM, Espinosa JC, Aguilar-Calvo P, Herva ME, Relano-Gines A, Villa-Diaz A, et al Elements modulating the prion species barrier and its passage consequences. PLoS One. 2014;9(3):e89722 doi: 10.1371/journal.pone.0089722 . 24608126 10.1371/journal.pone.0089722 PMC3946430

[17] Dawson M, Wells GAH, Parker BNJ, Scott AC. Primary Parenteral Transmission of Bovine Spongiform Encephalopathy to the Pig. Veterinary Record. 1990;127(13):338-. 2147795

[18] Konold T, Spiropoulos J, Chaplin MJ, Thorne L, Spencer YI, Wells GA, et al Transmissibility studies of vacuolar changes in the rostral colliculus of pigs. BMC Vet Res. 2009;5:35 Epub 2009/09/22. doi: 10.1186/1746-6148-5-35 . 19765298 10.1186/1746-6148-5-35 PMC2761866

[19] Ryder SJ, Hawkins SA, Dawson M, Wells GA. The neuropathology of experimental bovine spongiform encephalopathy in the pig. J Comp Pathol. 2000;122(2–3):131–43. Epub 2000/02/24. doi: 10.1053/jcpa.1999.0349 . 10684682 10.1053/jcpa.1999.0349

[20] Castilla J, Gutierrez-Adan A, Brun A, Doyle D, Pintado B, Ramirez MA, et al Subclinical bovine spongiform encephalopathy infection in transgenic mice expressing porcine prion protein. J Neurosci. 2004;24(21):5063–9. doi: 10.1523/JNEUROSCI.5400-03.2004 15163699 10.1523/JNEUROSCI.5400-03.2004 PMC6729370

[21] Espinosa JC, Herva ME, Andreoletti O, Padilla D, Lacroux C, Cassard H, et al Transgenic mice epressing porcine prion protein are resistant to cassical srapie but susceptible to seep bovine spongiform encephalopathy and atypical scrapie. Emerg Infect Dis. 2009;15(8):1214–21. doi: 10.3201/eid1508.081218 19751582 10.3201/eid1508.081218 PMC2815954

[22] Hedman C, Bolea R, Marin B, Cobriere F, Filali H, Vazquez F, et al Transmission of sheep-bovine spongiform encephalopathy to pigs. Vet Res. 2016;47:14 doi: 10.1186/s13567-015-0295-8 . 26742788 10.1186/s13567-015-0295-8 PMC4705642

[23] Andreoletti O, Morel N, Lacroux C, Rouillon V, Barc C, Tabouret G, et al Bovine spongiform encephalopathy agent in spleen from an ARR/ARR orally exposed sheep. J Gen Virol. 2006;87(Pt 4):1043–6. doi: 10.1099/vir.0.81318-0 . 16528056 10.1099/vir.0.81318-0

[24] Feraudet C, Morel N, Simon S, Volland H, Frobert Y, Creminon C, et al Screening of 145 anti-PrP monoclonal antibodies for their capacity to inhibit PrPSc replication in infected cells. J Biol Chem. 2005;280(12):11247–58. doi: 10.1074/jbc.M407006200 15618225 10.1074/jbc.M407006200

[25] Castilla J, Adan AG, Brun A, Pintado B, Ramirez MA, Parra B, et al Early detection of PRPres in BSE-infected bovine PrP transgenic mice. Arch Virol. 2003;148(4):677–91. doi: 10.1007/s00705-002-0958-4 12664293 10.1007/s00705-002-0958-4

[26] Lacroux C, Vilette D, Fernandez-Borges N, Litaise C, Lugan S, Morel N, et al Prionemia and leukocyte-platelet-associated infectivity in sheep transmissible spongiform encephalopathy models. J Virol. 2012;86(4):2056–66. doi: 10.1128/JVI.06532-11 . 22156536 10.1128/JVI.06532-11 PMC3302392

[27] Torres JM, Espinosa JC, Aguilar-Calvo P, Herva ME, Relano-Gines A, Villa-Diaz A, et al Elements modulating the prion species barrier and its passage consequences. PLoS One. 2014;9(3). 10.1371/journal.pone.0089722 PMC3946430 24608126

[28] Lacroux C, Comoy E, Moudjou M, Perret-Liaudet A, Lugan S, Litaise C, et al Preclinical detection of variant CJD and BSE prions in blood. PLoS Pathog. 2014;10(6):e1004202 Epub 2014/06/20. doi: 10.1371/journal.ppat.1004202 . 24945656 10.1371/journal.ppat.1004202 PMC4055790

[29] Moudjou M, Sibille P, Fichet G, Reine F, Chapuis J, Herzog L, et al Highly infectious prions generated by a single round of microplate-based protein misfolding cyclic amplification. MBio. 2014;5(1):e00829–13. Epub 2014/01/02. doi: 10.1128/mBio.00829-13 . 24381300 10.1128/mBio.00829-13 PMC3884057

[30] Seuberlich T, Zurbriggen A. Distinct molecular signature of bovine spongiform encephalopathy prion in pigs. Emerg Infect Dis. 2010;16(1):164 doi: 10.3201/eid1601.091104 . 20031075 10.3201/eid1601.091104 PMC2874374

[31] Hoffmann C, Eiden M, Kaatz M, Keller M, Ziegler U, Rogers R, et al BSE infectivity in jejunum, ileum and ileocaecal junction of incubating cattle. Veterinary Research. 2011;42. 10.1186/1297-9716-42-21 PMC3048543 21314904

[32] Kaatz M, Fast C, Ziegler U, Balkema-Buschmann A, Hammerschmidt B, Keller M, et al Spread of Classic BSE Prions from the Gut via the Peripheral Nervous System to the Brain. American Journal of Pathology. 2012;181(2):515–24. doi: 10.1016/j.ajpath.2012.05.001 22781833 10.1016/j.ajpath.2012.05.001

[33] Siso S, Jeffrey M, Steele P, McGovern G, Martin S, Finlayson J, et al Occurrence and cellular localization of PrPd in kidneys of scrapie-affected sheep in the absence of inflammation. J Pathol. 2008;215(2):126–34. doi: 10.1002/path.2336 . 18381605 10.1002/path.2336

[34] Wells GA, Hawkins SA, Austin AR, Ryder SJ, Done SH, Green RB, et al Studies of the transmissibility of the agent of bovine spongiform encephalopathy to pigs. J Gen Virol. 2003;84(Pt 4):1021–31. doi: 10.1099/vir.0.18788-0 . 12655106 10.1099/vir.0.18788-0

[35] Millson GC, Kimberlin RH, Manning EJ, Collis SC. Early distribution of radioactive liposomes and scrapie infectivity in mouse-tissues following administration by different routes. Vet Microbiol. 1979;4(2):89–99.

[36] Kimberlin RH. Unconventional ‘slow’ viruses. 8th edn ed London: Edward Arnold; 1990 671–93 p.

[37] Collinge J. Molecular neurology of prion disease. J Neurol Neurosurg Psychiatry. 2005;76(7):906–19. doi: 10.1136/jnnp.2004.048660 . 15965195 10.1136/jnnp.2004.048660 PMC1739714

[38] Houston F, McCutcheon S, Goldmann W, Chong A, Foster J, Siso S, et al Prion diseases are efficiently transmitted by blood transfusion in sheep. Blood. 2008;112(12):4739–45. doi: 10.1182/blood-2008-04-152520 18647958 10.1182/blood-2008-04-152520

[39] Castilla J, Saa P, Soto C. Detection of prions in blood. Nat Med. 2005;11(9):982–5. doi: 10.1038/nm1286 16127436 10.1038/nm1286

[40] Halliez S, Jaumain E, Huor A, Douet JY, Lugan S, Cassard H, et al White Blood Cell-Based Detection of Asymptomatic Scrapie Infection by Ex Vivo Assays. PLoS One. 2014;9(8). 10.1371/journal.pone.0104287 PMC4133197 25122456

[41] Lacroux C, Comoy E, Moudjou M, Perret-Liaudet A, Lugan S, Litaise C, et al Preclinical Detection of Variant CJD and BSE Prions in Blood. Plos Pathogens. 2014;10(6). 10.1371/journal.ppat.1004202 PMC4055790 24945656

[42] Kimberlin RH, Walker CA. Pathogenesis of experimental scrapie. Ciba Found Symp. 1988;135:37–62. . 3137002 10.1002/9780470513613.ch4

[43] Padilla D, Beringue V, Espinosa JC, Andreoletti O, Jaumain E, Reine F, et al Sheep and goat BSE propagate more efficiently than cattle BSE in human PrP transgenic mice. PLoS Pathog. 2011;7(3):e1001319 doi: 10.1371/journal.ppat.1001319 . 21445238 10.1371/journal.ppat.1001319 PMC3060172

[44] Plinston C, Hart P, Chong A, Hunter N, Foster J, Piccardo P, et al Increased susceptibility of human-PrP transgenic mice to bovine spongiform encephalopathy infection following passage in sheep. J Virol. 2011;85(3):1174–81. doi: 10.1128/JVI.01578-10 . 21084466 10.1128/JVI.01578-10 PMC3020518

[45] van Keulen LJ, Vromans ME, Dolstra CH, Bossers A, van Zijderveld FG. Pathogenesis of bovine spongiform encephalopathy in sheep. Arch Virol. 2008;153(3):445–53. doi: 10.1007/s00705-007-0007-4 . 18092124 10.1007/s00705-007-0007-4 PMC2249617

[46] McGovern G, Martin S, Jeffrey M, Bellworthy SJ, Spiropoulos J, Green R, et al Influence of breed and genotype on the onset and distribution of infectivity and disease-associated prion protein in sheep following oral infection with the bovine spongiform encephalopathy agent. J Comp Pathol. 2015;152(1):28–40. doi: 10.1016/j.jcpa.2014.09.004 . 25435510 10.1016/j.jcpa.2014.09.004

[47] Buschmann A, Groschup MH. Highly bovine spongiform encephalopathy-sensitive transgenic mice confirm the essential restriction of infectivity to the nervous system in clinically diseased cattle. J Infect Dis. 2005;192(5):934–42. doi: 10.1086/431602 . 16088845 10.1086/431602

[48] Eloit M, Adjou K, Coulpier M, Fontaine JJ, Hamel R, Lilin T, et al BSE agent signatures in a goat. Vet Rec. 2005;156(16):523–4. . 15833975 10.1136/vr.156.16.523-b

