# Supplementary figures and images for: Detection of PrPres in peripheral tissue in pigs with clinical disease induced by intracerebral challenge with sheep-passaged bovine spongiform encephalopathy agent
Source: PLoS One. 2018 Jul 5;13(7):e0199914. doi: 10.1371/journal.pone.0199914 (PMC6033439; doi:10.1371/journal.pone.0199914)

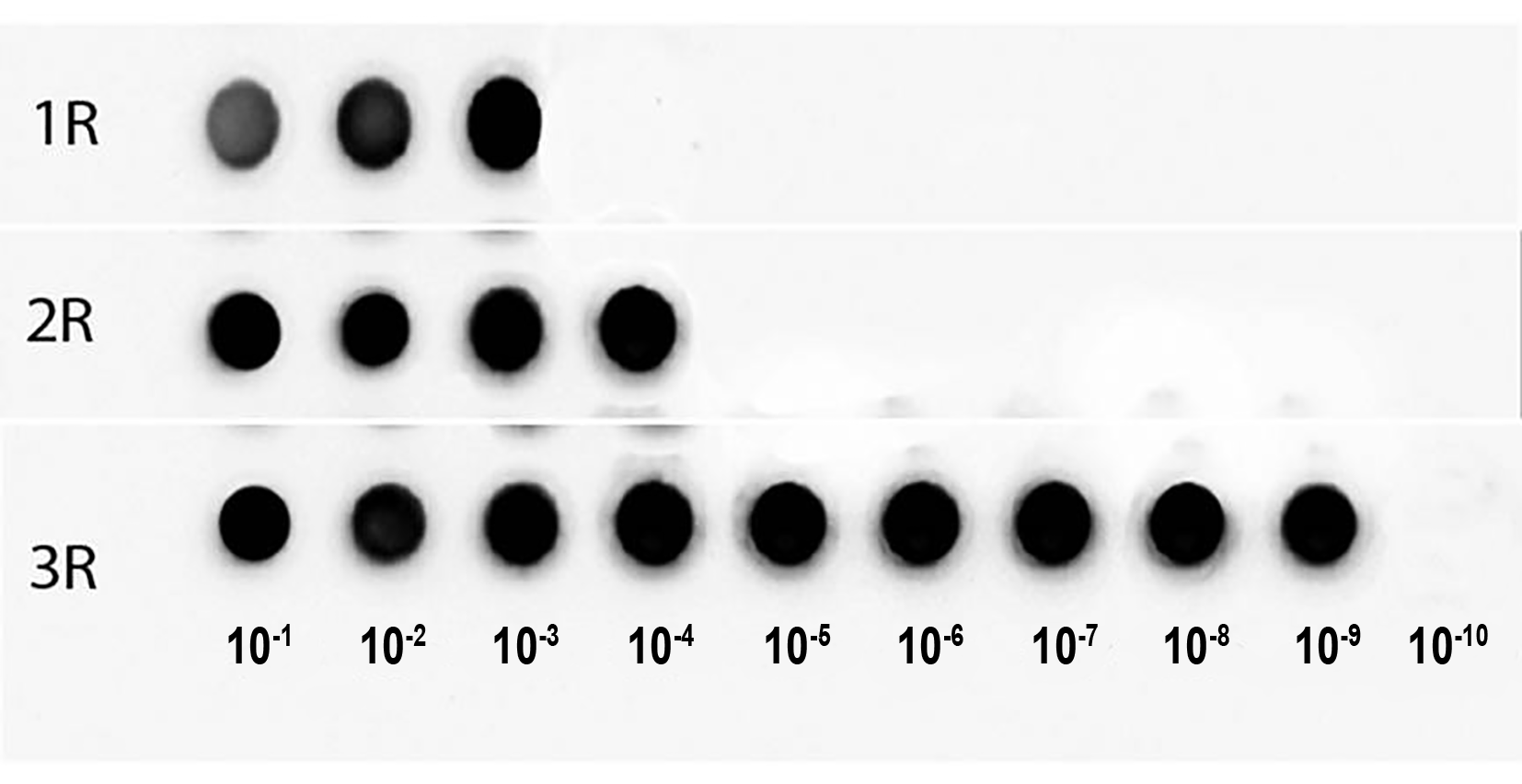

Supplement: S1 Fig — After 3 rounds of PMCA, PrPres was amplified in reactions seeded with a 10−9 dilution of a brain homogenate from the Sh-BSE-infected pig, demonstrating the sensitivity of the PMCA protocol used to detect pig Sh-BSE prions. The monoclonal Sha31 antibody was used for immunodetection. (TIF) [file pone.0199914.s001.tif]

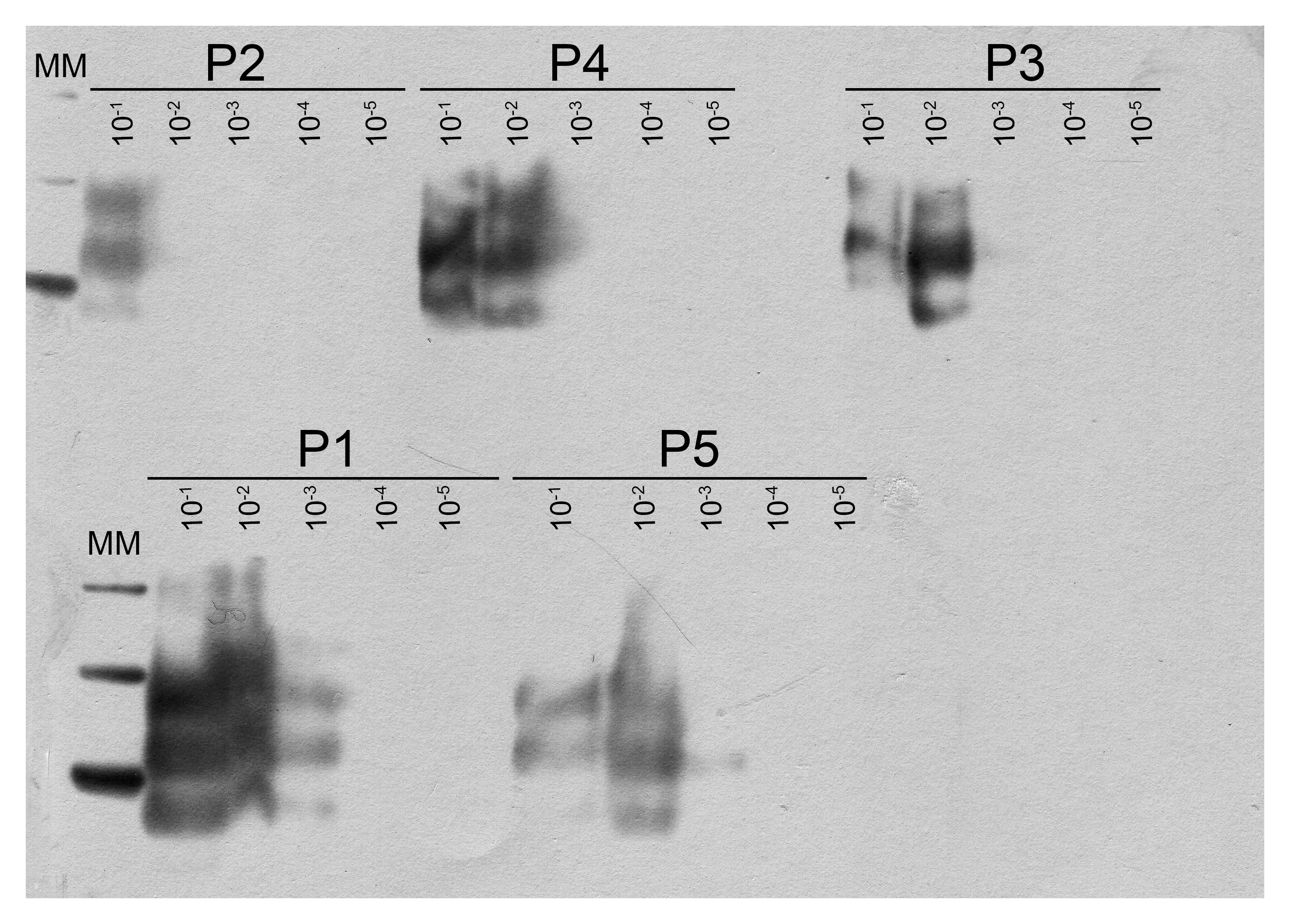

Supplement: S2 Fig — Western blot allowed detection of PrPres present in dilutions (10−1–10−3) of the original homogenates before PMCA. The monoclonal Sha31 antibody was used for immunodetection. MM: Magic Marker. (TIF) [file pone.0199914.s002.tif]
