# Supplementary material for: Detection of PrPres in peripheral tissue in pigs with clinical disease induced by intracerebral challenge with sheep-passaged bovine spongiform encephalopathy agent
Source: PLoS One. 2018 Jul 5;13(7):e0199914. doi: 10.1371/journal.pone.0199914 (PMC6033439; doi:10.1371/journal.pone.0199914)
Supplement: S1 Table — (PDF) [file pone.0199914.s003.pdf]

S1 Table: Individual survival periods and detection limits of Western Blot and PMCA for brain samples of Sh-BSE inoculated pigs

Sh-BSE inoculated pigs	Survival time (mpi)[a]	Max. Dilution WB (brain)	Max. Dilution PMCA(brain)[b]
P1	27	10^{-3}	10^{-9}
P2	30	10^{-1}	10^{-8}
P3	17	10^{-2}	10^{-9}
P4	26	10^{-2}	10^{-8}
P5	30	10^{-3}	10^{-9}

[a] Survival times are expressed as the number of months between inoculation and euthanasia. All inoculated animals developed clinical signs consistent with a TSE.

mpi Months postinoculation

[b] The detection limit of PMCA is expressed as the maximum dilution at which prion amplification was detected in brain samples after 3 rounds of PMCA
